# Allogeneic dendritic cells induce potent antitumor immunity by activating KLRG1^+^CD8 T cells

**DOI:** 10.1038/s41598-019-52151-3

**Published:** 2019-10-29

**Authors:** Chao Wang, Zhengyuan Li, Zhongli Zhu, Yijie Chai, Yiqing Wu, Zhenglong Yuan, Zhijie Chang, Zhao Wang, Minghui Zhang

**Affiliations:** 1 School of Medicine, Tsinghua University, Beijing, 100084 China; 2 Department of Clinical Laboratory, The Second Affiliated Hospital of Shandong First Medical University, Tai’an, Shandong 271000 China; 3 State Key Laboratory of Biomembrane and Membrane Biotechnology, School of Medicine, School of Life Sciences, Tsinghua University, Beijing, 100084 China; 4 Protein Science Key Laboratory of the Ministry of Education, School of Pharmaceutical Sciences, Tsinghua University, Beijing, 100084 China; 5 The Central Laboratory, The First Hospital of Tsinghua University, Beijing, 100084 China

**Keywords:** Immunization, Cell vaccines

## Abstract

The graft-versus-leukemia effect reminds us to observe the allogeneic cell elicited anti-tumor immune responses. Here we immunized recipient B6 mice with different types of allogenic leukocytes and found that vaccination with allogenic dendritic cells (alloDC) elicited the most efficient protection against broad-spectrum tumors. The recipient lymphocytes were analyzed and the data showed that CD8 T cells increased significantly after immunization and expressed effector memory T cell marker KLRG1. Functional evaluation demonstrated that these KLRG1^+^CD8 T cells could kill tumor cells *in vitro* and *in vivo* in Granzyme B- and Fas/FasL-dependent manners with no tumor antigen specificity, and tend to migrate into tumor sites by high expression of heparanase. Adoptive transfer of these cells could provide antitumor protection against tumors. AlloDC could also treat mice with residual tumors and combination of anti-PD1 antibody could enhance this effects. Together, our study showed that alloDC-immunization could induce potent antitumor effect through the expansion of KLRG1^+^CD8 T cells, which can work as both preventive and therapeutic tumor vaccines.

## Introduction

The ability of allogeneic cells to exert an antitumor effect has been observed since allogeneic bone marrow transplantation (allo-BMT) was developed as one of the few treatments for drug-resistant hematological malignancies^1–3^. Investigations on the mechanisms of this graft-versus-leukemia (GVL) effect demonstrated that host antigen presenting cells could present minor histocompatibility antigen or tumor antigen to activate infused donor lymphocytes, which mediated the elimination of cancer cells^1,3^. T cells were shown to play key roles in graft-versus-tumor effects^3–5^. And NK cells also contributed to antitumor effects when T cell-depleted hematopoietic stem cell transplantation was performed^5,6^.

The graft-versus-leukemia effect provides an example of the allogeneic cell-stimulated immune system recognizing and eliminating tumor cells. In the past decades, accumulating evidence has showed that allogeneic cells could trigger strong antitumor immunity *in vivo*. Allogeneic GM-CSF-secreting tumor cells have been found to induce tumor antigen specific CD8 T cells when used as tumor vaccines, although these allogeneic tumor cells only provided the source of tumor antigens and GM-CSF^7–9^. Tumor antigen loaded allogeneic dendritic cells also showed comparable capability with autologous dendritic cells to elicit tumor antigen specific CD8 T cells and thus provide antitumor protection^10–13^. Besides, allogeneic fibroblasts loaded with tumor antigens could be cross-presented by host dendritic cells, by which tumor antigen specific CD8 T cell responses could also be stimulated^14^. In these findings, allogeneic antigen presenting cells were usually used as the substitution of corresponding autologous cells for convenient and reliable purpose and loaded with tumor antigens to elicit CTL-mediated antitumor effects. Recently, immunologists found that allogeneic dendritic cells could deliver tumor antigens to bystander autologous dendritic cells, which then activated tumor antigen specific immune responses against tumors^15,16^. However, whether and how allogeneic dendritic cells themselves could allostimulate recipient T cells was still unclear.

It is noteworthy that in addition to these CTL-mediated antigen specific immune responses, allogenic cells could also elicit non-specific immune responses by allogeneic MHC molecules^17^. For example, infused NK cells could recognize allogeneic MHC molecules via NK cell receptors and kill tumor cells^6,18,19^. More important, about 1~10% of total T cells could also be primed by the foreign MHC molecules^17,20^. What is the function of these T cells? Are they involved in antitumor effects? If so, what is the responsible effector lymphocyte population? What is their immunological features and how do they function in antitumor effects? These questions remain to be elucidated.

In this study, we immunized B6 mice (H-2^b^) with splenocytes from DBA mice (H-2^d^) and observed a potent broad-spectrum antitumor effect in B6 mice, demonstrating that allogeneic cells could elicit tumor antigen independent antitumor responses in recipient individuals. Further investigation showed that allogeneic dendritic cells (alloDC) could prime the most efficient antitumor effects and induce expansion of KLGR1^+^CD8 T cells which are the main effector cells in the immune responses against tumors and exert their anti-tumor effects via non-specific cytotoxicity. The results demonstrated that allogenic DC immunization or adoptive transfer of expanded KLGR1^+^CD8 T cells might provide a potential strategy for tumor treatment.

## Results

### Allogeneic dendritic cells could elicit efficient antitumor effects

**Figure 1:**
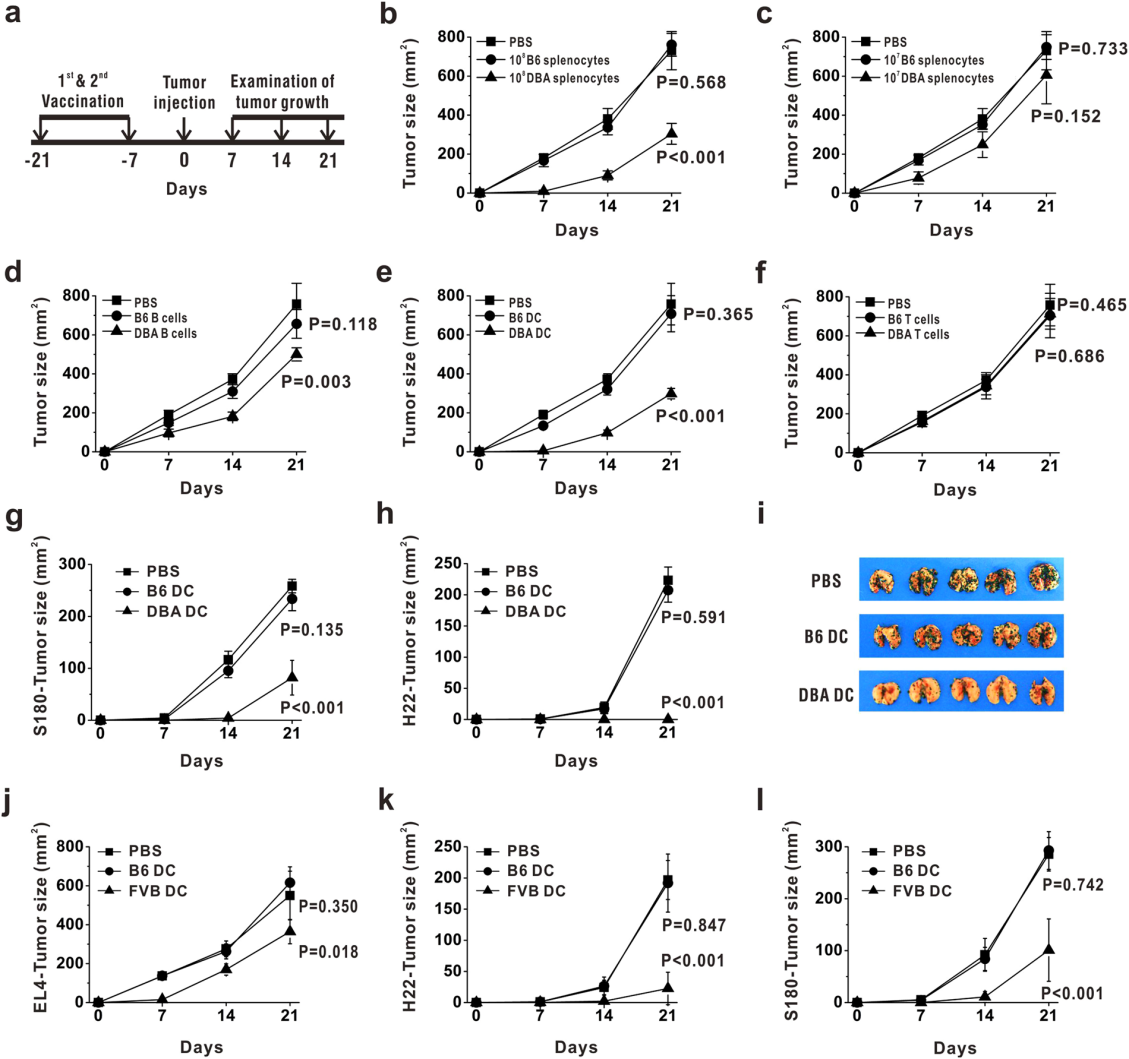
Allogeneic dendritic cells could elicit efficient antitumor effects. (**a**) Schematic diagram of the investigation of antitumor effects by allogeneic immunocytes. (**b**,**c**) B6 mice were pre-immunized with 10^8^ or 10^7^ of splenocytes from DBA/2 mice (▲) or B6 mice (●), respectively. After immunization for two times, they were inoculated subcutaneously with 2 × 10^6^ EL4 cells. EL4 tumor sizes were detected at indicated time points. (d-f) 5 × 10^7^ TCRβ^−^CD19^+^B cells (**d**), 1 × 10^6^ TCRβ^−^CD19^−^CD11c^+^Ia^+^dendritic cells (**e**) and 3 × 10^7^ TCRβ^+^CD19^−^T cells (**f**) were isolated from splenocytes of DBA/2 (▲) or B6 (●) mice by flow cytometry and injected intraperitoneally into recipient B6 mice for two times. Recipient mice were inoculated subcutaneously with 2 × 10^6^ EL4 cells. EL4 tumor sizes were detected at indicated time points. (**g**–**i**) B6 mice were pre-immunized intraperitoneally by 1 × 10^6^ DBA DC (▲) or B6 DC (●) for two times. Then mice were inoculated subcutaneously with 1 × 10^6^ S180 cells (**g**) or 1 × 10^6^ H22 cells (**h**), or injected intravenously with 5 × 10^5^ B16 cells (**i**), and tumor growth were monitored. (**j**–**l**) DCs from FVB mice (▲) or B6 mice (●) were immunized into B6 mice for two times. Then recipient mice were inoculated subcutaneously with 2 × 10^6^ EL4 cells (**j**), 1 × 10^6^ H22 cells (**k**) or 1 × 10^6^ S180 cells. (**l**) Tumor sizes were recorded at indicated time points. Recipient mice injected with PBS (■) were used as the blank control. These experiments were repeated for 3 times. n = 5 for each repeat. P values indicated the statistical significance when comparing with blank control group (ip PBS group).

B6 mice (H-2^b^ background) were pre-immunized with isolated splenocytes from donor DBA/2 mice (H-2^d^ background) or B6 mice twice and then was subcutaneously inoculated with EL4 thymoma cells (as was scheduled in Fig. 1a). We found that immunization of B6 mice with 10^8^ splenocytes from DBA/2 mice could efficiently inhibit EL4 tumor growth, while vaccination with the same amount of autologous splenocytes had no effects (Fig. 1b). B6 mice immunized with 3 × 10^7^ splenocytes from DBA/2 mice could also acquire antitumor protection although weaker than immunized with 10^8^ cells (Supplementary Fig. 1). But when we immunized B6 mice with 10^7^ splenocytes from DBA/2 mice, antitumor effects were not observed (Fig. 1c), indicating a dose-dependent allogeneic splenocyte-elicited antitumor immune response. To find out which cell subset played a key role in inducing antitumor immunity, we compared 3 × 10^7^ T cells (TCRβ^+^CD19^−^), 5 × 10^7^ B cells (TCRβ^−^CD19^+^) as well as 1 × 10^6^ DCs (TCRβ^−^CD19^−^CD11c^+^Ia^+^), respectively, from DBA/2 mice with those from B6 mice, in their antitumor efficacy as tumor vaccines. The data showed that both B cells (Fig. 1d) and DCs (Fig. 1e) from DBA/2 mice exhibited notable inhibition of tumor growth, of which DCs were more efficient. Neither allogeneic T cells (Fig. 1f) nor autologous T cells, B cells or DCs could elicit antitumor capability. These data demonstrated that DCs and B cells were responsible for the antitumor effects elicited by allogeneic splenocytes. As alloDC showed the most potent antitumor effects, immunization with alloDC was exploited as the ideal model to study the underlying mechanisms.

To confirm the alloDC-elicited antitumor effects, S180 sarcoma cells (Fig. 1g), H22 hepatocarcinoma cells (Fig. 1h) or B16 melanoma cells (Fig. 1i) were also inoculated into DBA DC-vaccinated, B6 DC-vaccinated or PBS-injected B6 mice, respectively. Consistent with the data in Fig. 1e, these data showed that immunization with DBA DC, but not B6 DC, could efficiently inhibit the tumor growth in recipient B6 mice (Fig. 1g–i). To exclude the donor strain specificity of alloDC-elicited antitumor effects, we used dendritic cells from FVB/NCrlVr mice (H-2^q^) to immunize B6 mice, and tested their tumor inhibition capability. Our data presented that the tumor growth of EL4 thymoma cells (Fig. 1j), H22 hepatocarcinoma cells (Fig. 1k) and S180 sarcoma cells (Fig. 1l) could be significantly suppressed in FVB DC-vaccinated mice, but not in B6 DC-vaccinated mice, demonstrating that the broad-spectrum antitumor effects elicited by alloDC is universal phenomenon.

### CD8 T cells were involved in alloDC-elicited antitumor effects

**Figure 2:**
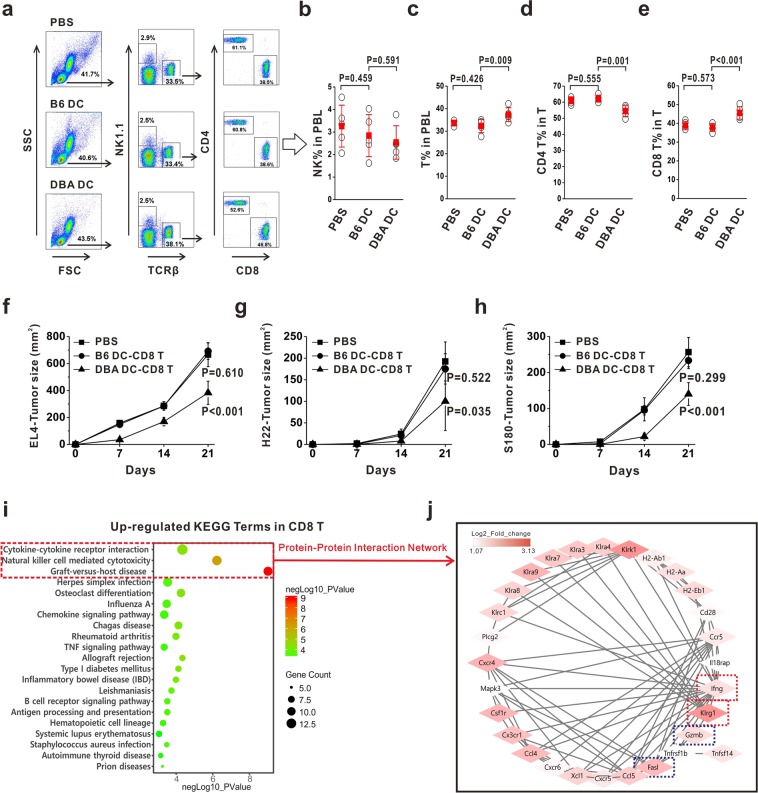
CD8 T cells were involved in alloDC-elicited antitumor effects. (**a**–**e**) Peripheral blood was collected from DBA DC-, B6 DC- or PBS-injected mice and main lymphocyte subsets involved in antitumor immunity were examined. Proportions of indicated subsets were statistically compared among the three groups. (**f**–**h**) B6 mice were pre-injected with 1 × 10^7^ CD8 T cells from DBA DC-immunized mice (▲, labeled as DBA DC-CD8 T), B6 DC-immunized mice (●, labeled as B6 DC-CD8 T) or PBS (■). Then recipient mice were inoculated with 2 × 10^6^ EL4 cells (**f**), or 1 × 10^6^ H22 cells (**g**) or 1 × 10^6^ S180 cells (**h**). Tumor growth curves were depicted and compared among the three groups. These experiments were repeated thrice, where n = 5 for each repeat. P values indicated the statistical significance when comparing with blank control group (PBS group). (**i**,**j**) RNAseq on CD8 T cells from autoDC- and alloDC-vaccinated mice was performed and their DEGs were analyzed using R language and Cytoscape software. KEGG pathway enrichment of DEGs (**i**) and protein-protein interaction network analysis focusing on genes in the three most significantly enriched pathways (**j**) was demonstrated. Significantly enriched antitumor-associated pathways and molecules were highlighted.

To figure out the mechanisms underlying how alloDC could elicit antitumor effects, peripheral blood lymphocytes (PBL) from B6 mice immunized with DBA DC were collected to analyze the subset proportions by flow cytometry (Fig. 2a). The data showed that the proportions of NK cells did not show a significant change between DBA DC- and B6 DC-immunized mice (Fig. 2b), the proportions of total T cells increased from 32.3 ± 2.9% in B6 DC-immunized mice to 37.3 ± 3.1% in DBA DC-immunized mice (Fig. 2c). Of these T cells, the proportion of CD4 T cells in total T cells declined from 61.2% ± 3.1% in B6 DC-immunized mice to 52.7 ± 2.3% in DBA DC-immunized mice (Fig. 2d), while the proportion of CD8 T cells increased from 37.8% ± 2.1% in B6 DC-immunized mice to 45.5 ± 3.3% in DBA DC-immunized mice (Fig. 2e). These data suggested that alloDC-vaccination could activate CD8 T cells significantly, implying their possible involvement in antitumor effects. To investigate it, we adoptively transferred these alloDC-activated CD8 T cells into B6 mice, which were inoculated with EL4 (Fig. 2f), H22 (Fig. 2g) or S180 (Fig. 2h) tumor cells. The data showed that only infusion of CD8 T cells from DBA DC-vaccinated mice, but not from B6 DC-vaccinated mice, could inhibit tumor growth (Fig. 2f–h). This indicated that CD8 T cells from alloDC-immunized mice, but not from autologous DC (autoDC)-immunized mice, could provide an efficient antitumor protection.

To further explore the reason why alloDC-activated CD8 T cells could have the antitumor capability, we compared the transcriptomic profiles of CD8 T cells from alloDC-vaccinated mice with those from autoDC-vaccinated mice and identified 259 up-regulated differentially expressed genes (DEGs). After performing KEGG enrichment analysis on these genes, we found that the three pathways were enriched with greatest significance, which were cytokine-cytokine receptor interaction, natural killer cell mediated cytotoxicity and graft-versus-host disease (Fig. 2i). Protein-protein interaction network analysis of DEGs in these three pathways demonstrated that these CD8 T cells expressed effector associated molecules, including IFN-gamma, Granzyme B, FasL, KLRG1 and other NK cell receptors, of which KLRG1 was identified to be the most significant marker (Fig. 2j). We also compared the transcriptomic profiles of CD4 T cells and NK cells respectively between auto- and allo-DC vaccinated mice, but found that much fewer notable antitumor associated molecules were enriched in KEGG enriched pathways as well as protein-protein interaction networks (Supplementary Fig. 2), implying that CD4 T cells and NK cells might play less essential roles than CD8 T cells in alloDC-elicited antitumor effects.

### KLRG1^+^CD8 T cells were involved in alloDC-vaccinated tumor resistant B6 mice

**Figure 3:**
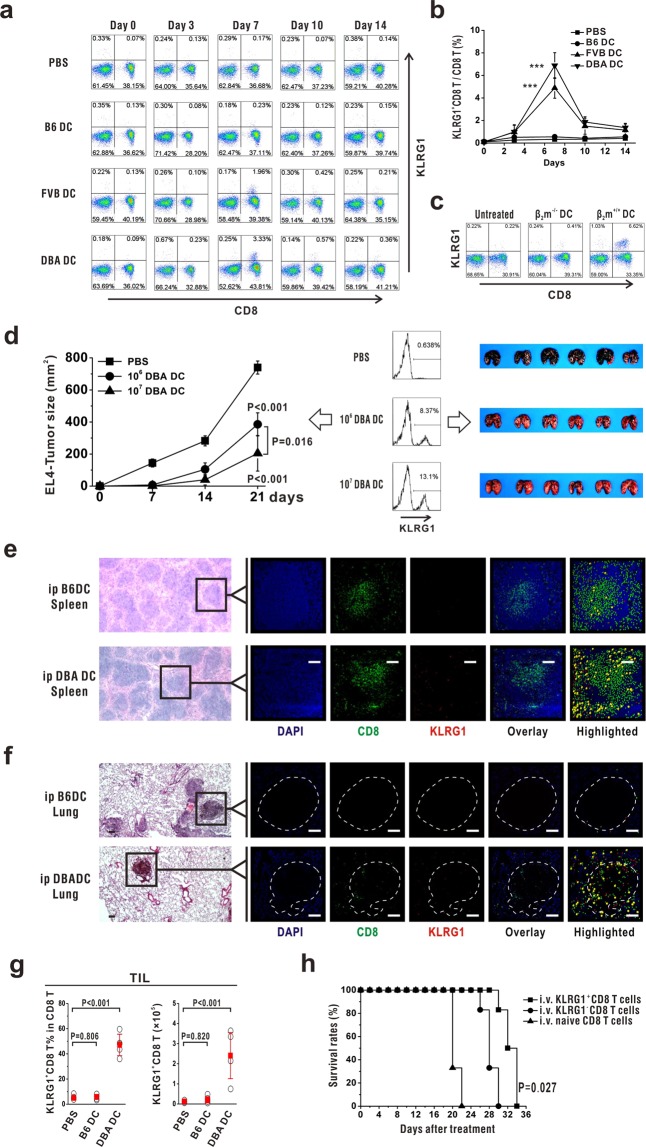
KLRG1^+^CD8 T cells were involved in alloDC-elicited antitumor effects. (**a**,**b**) B6 mice were immunized with B6 DC, FVB DC or DBA DC. Then percentages of KLRG1^+^CD8 T cells in peripheral blood lymphocytes were detected at indicated time points using flow cytometry. Statistical data were shown in (**b**). (**c**) DBA/2 mice were immunized with either β_2_m^−/−^ DC or B6 DC. Then percentages of KLRG1^+^CD8 T cells were detected on day 7. (**d**) B6 mice were pre-vaccinated by different doses of DBA DC and then inoculated with EL4 subcutaneously or B16 intravenously. Peripheral KLRG1^+^CD8 T cells were analyzed by flow cytometry and their tumor growth was shown. (**e**) Frozen 8-μm sections were processed from B6 DC- or DBA DC-immunized spleens and were stained with DAPI (blue), PE labeled anti-CD8 (green) and APC labeled anti-KLRG1 (red). In the highlighted images, green balls represent cells only expressing CD8, while red balls represent cells only expressing KLRG1. When both CD8 and KLRG1 were expressed on the same cells, they were highlighted by a larger yellow ball. The length of the scale bar is 100 μm. (**f**) Frozen 8-μm sections from tumor-bearing lungs were stained with DAPI (blue), PE labeled anti-CD8 (green) and APC labeled anti-KLRG1 (red). The dotted white line shows the tumor border. The length of the scale bar is 100 μm. (**g**) Metastatic lung tissue was harvested and the proportions and absolute numbers of KLRG1^+^CD8 T cells were compared among the mice pre-immunized by PBS, B6 DC and DBA DC. (h) 1 × 10^6^ KLRG1^+^CD8 T cells, KLRG1^−^CD8 T cells as well as naïve CD8 T cells were sorted from DBA DC-immunize B6 mice and adoptive transferred intravenously into B6 mice injected with 1 × 10^4^ B16 cells. Survival rates of each group were recorded and shown. These experiments were repeated for 3 times. n = 6 for each repeat. Error bars show standard deviation. P values indicated the statistical significance when comparing with blank control group (PBS group or naive CD8 T group).

As KLRG1 expression was significantly elevated in DBA DC-activated CD8 T cells, the population of KLRG1^+^CD8 T cells were then detected in alloDC-vaccinated mice. We found that both allogeneic FVB DC and DBA DC could efficiently stimulate the increase of KLRG1^+^CD8 T cells, while autologous B6 DC could not (Fig. 3a). Dynamic changes of the proportions of KLRG1^+^CD8 T cells after immunization were depicted (Fig. 3b), which also showed their significant increases in alloDC-vaccinated mice, but not autoDC-vaccinated mice. Therefore, we could conclude that allogeneic MHC molecules could activate the population of KLRG1^+^CD8 T cells. But when we immunized DBA/2 mice with MHC-I deficient DC from β_2_m^−/−^ mice or wild-type B6 DC, the data showed that only MHC-I competent B6 DC, but not MHC-I deficient DC, could activated KLRG1^+^CD8 T cells in DBA/2 mice (Fig. 3c). Since DC from β_2_m^−/−^ mice expressed competent allogenic MHC class II molecules but failed to induced expansion of KLRG1^+^CD8 T cells (Fig. 3c, the middle panel), we concluded that allogeneic MHC-I molecules played an essential role in this model.

To validate the involvement of KLRG1^+^CD8 T cells in alloDC-vaccinated antitumor effects, we collected PBL from PBS-, 10^6^ DBA DC- or 10^7^ DBA DC-vaccinated B16-bearing mice. The data showed that a greater number of KLRG1^+^CD8 T cells and a more efficient antitumor capability was found in 10^7^ DBA DC-immunized mice than in 10^6^ DBA DC-immunized mice (Fig. 3d), demonstrating a close correlation among alloDC vaccination, KLRG1^+^CD8 T cell activation and antitumor capability acquisition. To provide an intuitive view, we performed immunofluorescent staining, demonstrating more CD8 and KLRG1 double positive T cells (yellow) in DBA DC-immunized spleens (Fig. 3e). Immunofluorescent staining in B16 lung metastatic also showed that more KLRG1^+^CD8 T cells (yellow) infiltration into tumor sites (Fig. 3f) in DBA DC-vaccinated mice. Statistical data also presented that both the proportions and numbers of tumor infiltrating KLRG1^+^CD8 T cells was increased in DBA DC, but not B6 DC-vaccinated mice (Fig. 3g). Adoptive transfer of KLRG1^+^CD8 T cells showed a stronger protection against B16 melanoma cells than KLRG1^−^CD8 T cells (Fig. 3h). All these data suggested that KLRG1^+^CD8 T cells were involved in the antitumor effects in alloDC-vaccinated wild type B6 mice.

### Immunological characteristics of KLRG1^+^CD8 T cells

**Figure 4:**
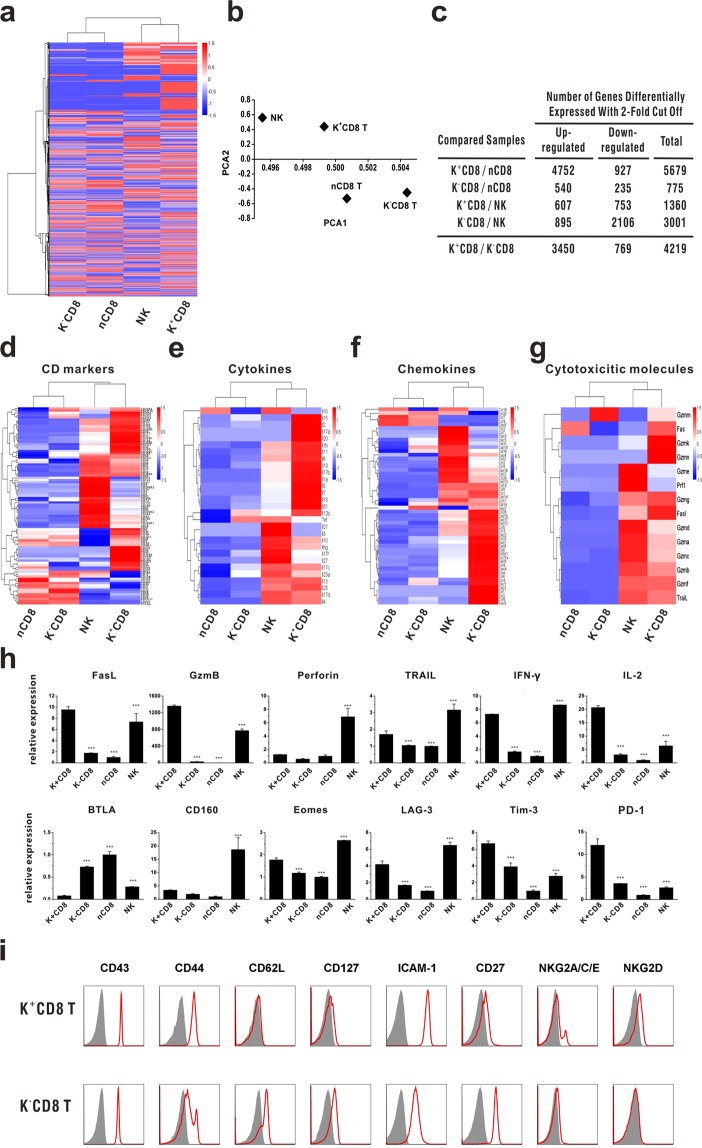
Gene expression profile of KLRG1^+^CD8 T cells. RNA-seq analysis was performed between KLRG1^+^CD8 T cells and KLRG1^−^CD8 T cells at day 3 after 2nd immunization. NK cells from the same immunized mice and naïve CD8 T cells were used as controls. (**a**,**b**) Relative intensities of all genes in KLRG1^+^CD8 T cells and KLRG1^−^CD8 T cells compared with naive CD8 T cells and NK cells are plotted as heat maps (**a**) or PCA plots (**b**) to display the relationship between various populations. (**c**) Total number of genes differentially expressed (up- or down-regulated; 2-fold cutoff) between the indicated comparison groups is shown. (**d**–**g**) Expression profiles of immunity-related genes, including CD surface markers (**d**), interleukins (**e**), chemokine and chemokine receptors (**f**), and cytotoxicity-associated molecules (**g**), were compared among indicated subsets. (**h**) Real-time PCR analysis of effector-associated markers was performed. ^***^Means P < 0.001 compared with KLRG1^+^CD8 T cells^.^ (**i**) Some T cell activation markers and NK cell markers were compared between KLRG1^+^CD8 T cells and KLRG1^−^CD8 T cells by flow cytometry. Real-time PCR and flow cytometry were repeated thrice.

To further characterize the population of KLRG1^+^CD8 T cells in alloDC activated antitumor model, we performed a genome-wide transcriptome sequencing of KLRG1^+^CD8 T cells and KLRG1^−^CD8 T cells at day 3 after 2^nd^ immunization. NK cells from the same immunized mice and naïve CD8 T cells were used as controls. Relative intensities of all genes in KLRG1^+^CD8 T cells, KLRG1^−^CD8 T cells, naive CD8 T cells and NK cells were plotted as heat maps (Fig. 4a) or PCA plots (Fig. 4b) to depict the relationship between these populations. Total number of genes differentially expressed (up- or down-regulated; 2-fold cut off) between the indicated comparison groups was also shown (Fig. 4c). The data demonstrated that the gene expression profile of KLRG1^+^CD8 T cells was closer with that of NK cells, while the gene expression profile of KLRG1^−^CD8 T cells was more similar with that of naïve CD8 T cells. In order to fully clarify the immunological characteristics of KLRG1^+^CD8 T cells, we depicted their expression of surface CD makers (Fig. 4d), cytokines (Fig. 4e), chemokine and chemokine receptors (Fig. 4f) and cytotoxicity associated molecules (Fig. 4g) among KLRG1^+^CD8 T cells, KLRG1^−^CD8 T cells, NK cells and naïve CD8 T cells. These four subsets could also be divided into two groups according to cluster analysis of their expression profiles. One included KLRG1^+^CD8 T cells and NK cells, while the other included KLRG1^−^CD8 T cells and naïve CD8 T cells. Compared with KLRG1^−^CD8 T cells, KLRG1^+^CD8 T produced more kinds of cytokines (Fig. 4e), such as IL-2 and IFN-gamma, which promoted antitumor immune responses. KLRG1^+^CD8 T cells also expressed high amounts of chemokine and chemokine receptors (Fig. 4f), which is consistent with their higher migration capacity into tumor sites. Besides, the expression of cytotoxicity-associated molecules, including granzyme, perforin, Fas/Fas Ligand and TRAIL, was higher in KLRG1^+^CD8 T cells than in KLRG1^−^CD8 T cells (Fig. 4g), suggesting that KLRG1^+^CD8 T cells might have higher cytotoxicity against tumor cells. Real-time PCR analysis of selected functional molecules showed a good consistence with RNA-seq data (Fig. 4h), which also suggested that KLRG1^+^CD8 T cells expressed higher levels of some inhibitory checkpoint molecules, such as Lag3, Tim-3 and PD-1 (Fig. 4h). We also detected T and NK cell function associated surface markers, and the data showed that these KLRG1^+^CD8 T cells were TCRβ^+^CD43^hi^CD44^hi^CD27^low^CD127^low^ CD62L^low^ cells with expression of some NK receptors (Fig. 4i). Considering that activated CD8 T cells could upregulate expressions of some NK cell markers, these KLRG1^+^CD8 T cells have the phenotype of activated effector CD8 T cells, indicating their tumoricidal function like effector CD8 T cells.

### KLRG1^+^CD8 T cells kill tumor cells via Granzyme B or FasL pathway

**Figure 5:**
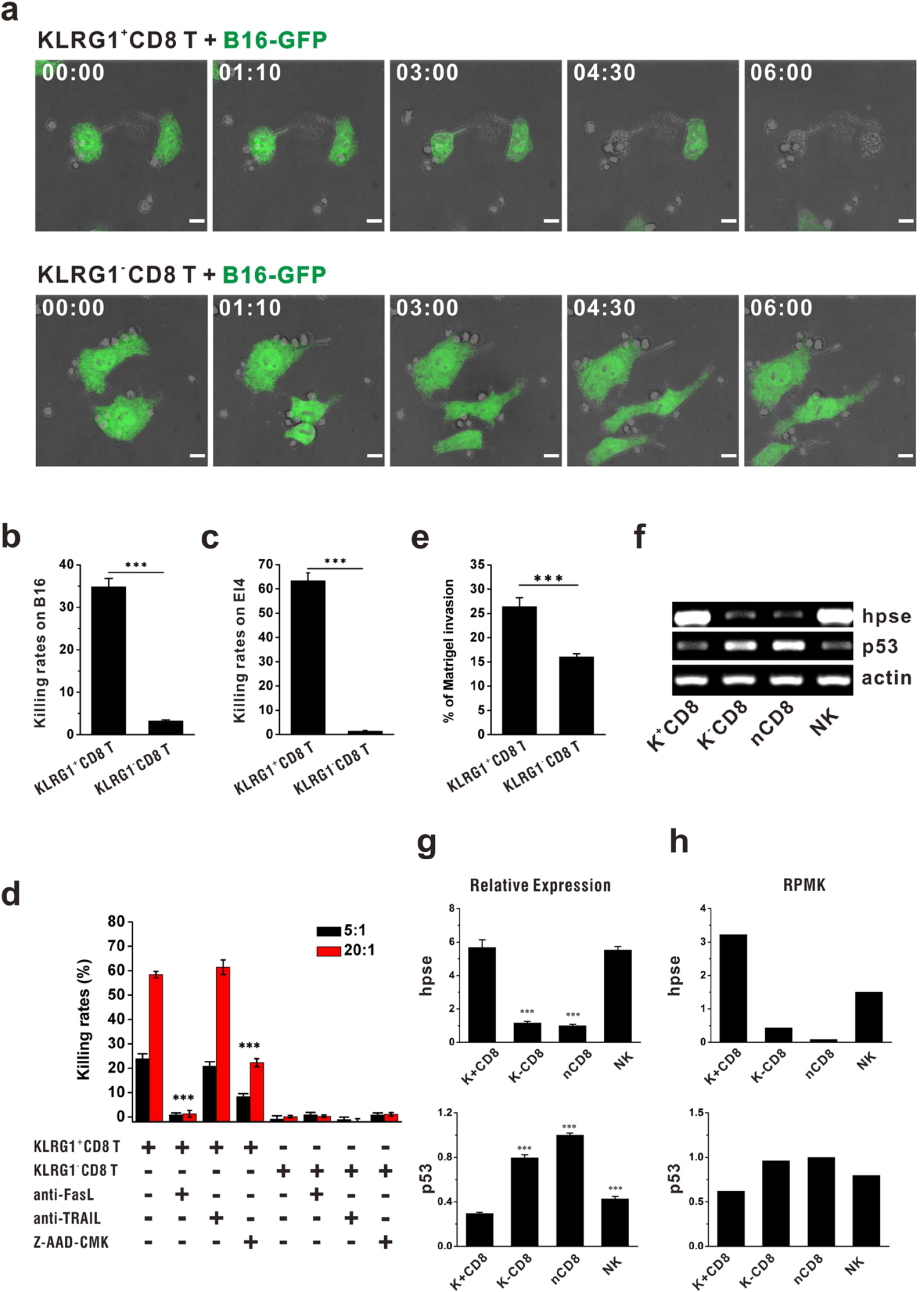
Mechanisms for KLRG1^+^CD8 T cells suppressing tumors. (**a**) KLRG1^+^CD8 T cells or KLRG1^−^CD8 T cells were co-cultured with B16-GFP cells (green) at the E:T ratio of 5:1, and the killing process was captured by PE spinning disk live cell confocal microscope with a 60 × oil immersion lens. (**b**) KLRG1^+^CD8 T cells or KLRG1^−^CD8 T cells were co-cultured with B16-GFP cells at the E:T ratio of 5:1 for 24 hours. Then target cells were collected and stained with 7-AAD. Percentages of 7-AAD positive populations indicated the killing rates. (**c**) KLRG1^+^CD8 T cells or KLRG1^−^CD8 T cells were co-cultured with EL4 cells at the E:T ratio of 20:1 for 12 hours. Then target cells were collected and stained with 7-AAD. Percentages of 7-AAD positive populations indicated the killing rates. (**d**) KLRG1^+^CD8 T cells and KLRG1^−^CD8 T cells were co-cultured with EL4 cells at the E:T ratio of 5:1 and 20:1 for 24 h with or without 50ug/mL anti-FasL, 50ug/mL anti-TRAIL, and 50 μM Granzyme B inhibitor Z-AAD-CMK. Cytotoxicity against target cells was evaluated and shown. (**e**) In an *in vitro* matrigel invasion experiment, KLRG1^+^CD8 T cells or KLRG1^−^CD8 T cells were sorted and inoculated on the upper layer. After 24 hours, penetrated cells on the lower layer were collected and calculated. (**f**–**h**) Real-time PCR (**f**,**g**) was carried out to examine the gene expression of heparanase and p53, which were also confirmed by RNA-seq analysis. (**h**) *In vitro* experiments were performed in triplicates for three times.

Since their higher expression of cytotoxicity associated molecules (Fig. 4g), KLRG1^+^CD8 T cells were co-cultured with B16 cells to evaluate their cytotoxicity. As shown in Fig. 5a, live imaging data showed that KLRG1^+^CD8 T cells could kill GFP transgenic B16 cells (B16-GFP cells) within 6 hours (Supplementary Video 1), while KLRG1^−^CD8 T cells could not kill B16-GFP cells (Supplementary Video 2). Statistic data also showed that compared with KLRG1^−^CD8 T cells, which did not display obvious cytotoxicity against tumor cells, KLRG1^+^CD8 T cells killed B16 cells (Fig. 5b) and EL4 cells (Fig. 5c) with much higher killing rates. Cytotoxicity mediated by lymphocytes is usually attributed to either the granule exocytosis pathway, or the Fas/FasL pathway^21^, or the TRAIL pathway^22^. Therefore, we tried to suppress the cytotoxicity of KLRG1^+^CD8 T cells by addition of Granzyme B inhibitor, Z-AAD-CMK, or anti-FasL neutralizing antibody, or anti-TRAIL neutralizing antibody. The data presented that inhibiting Granzyme B or FasL could efficiently abolish their killing effects, whereas neutralizing TRAIL did not have an affect (Fig. 5d). Our data have shown that more KLRG1^+^CD8 T cells could migrate and infiltrate into tumor sites than KLRG1^−^CD8 T cells (Fig. 3f). In an *in vitro* matrigel invasion experiment, we further showed that KLRG1^+^CD8 T cells could penetrate the matrigel more efficiently than KLRG1^−^CD8 T cells (Fig. 5e). It was reported that the invasive capability of effector T cells was associated with the expression of heparanase^23^. Therefore, real-time PCR was carried out to examine the expression levels of heparanase and its negative regulator p53. The data showed that compared with KLRG1^−^CD8 T cells, KLRG1^+^CD8 T cells expressed a higher level of heparanase but a lower level of p53 (Fig. 5f,g), which was then confirmed by sequencing data (Fig. 5h). Therefore, compared with KLRG1^−^CD8 T cells, higher expression of heparanase might contribute to the migration of KLRG1^+^CD8 T cells into tumor sites, where KLRG1^+^CD8 T cells could exert stronger cytotoxicity against tumor cells in FasL- and Granzyme B-dependent manners.

### AlloDCs act as therapeutic vaccine to treat residual cancer

**Figure 6:**
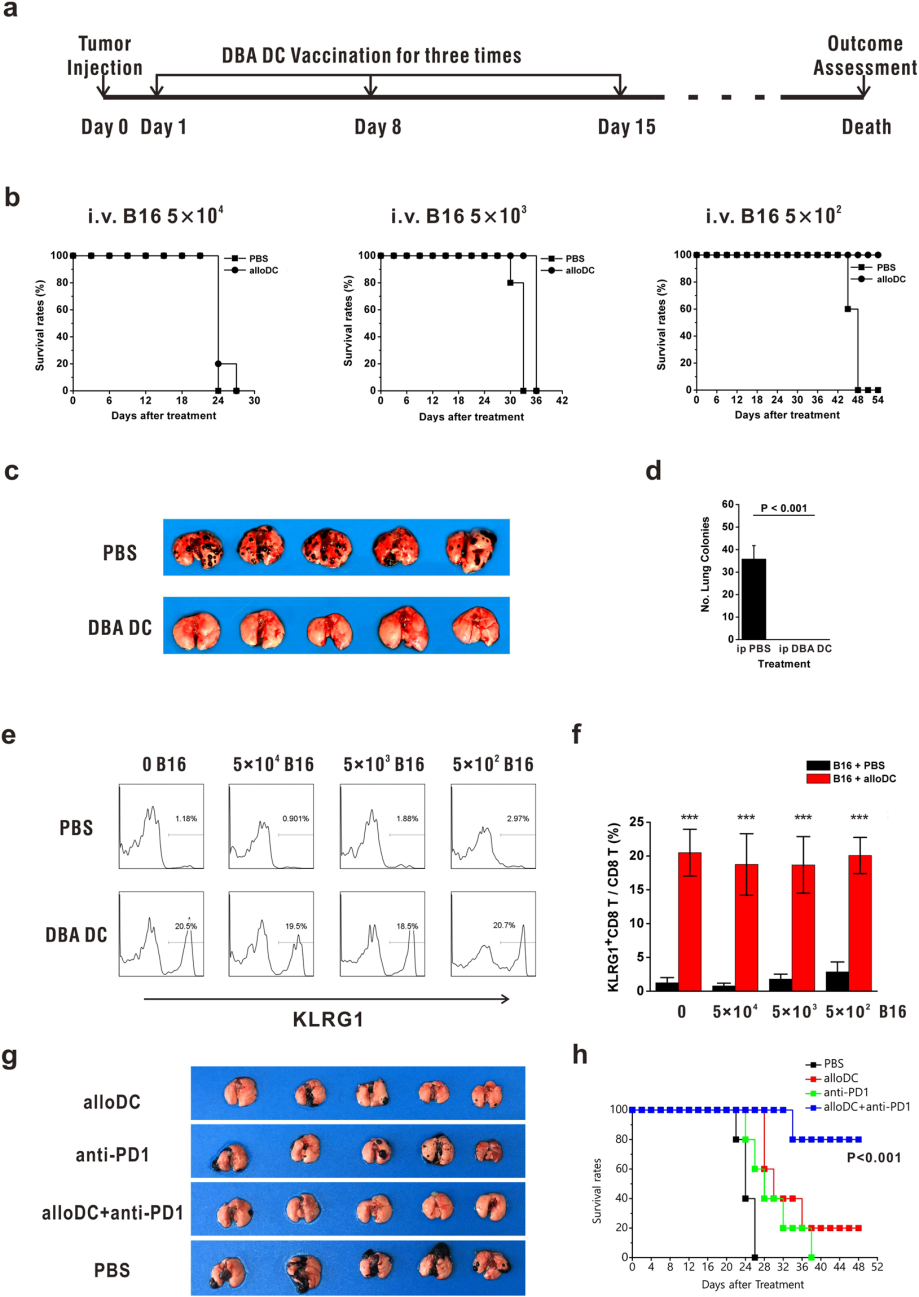
AlloDC as therapeutic vaccine to treat residual cancer. (**a**) Schematic diagram of exploiting alloDC to treat mice with residual B16 cells. (**b**) 5 × 10^4^, 5 × 10^3^ or 5 × 10^2^ B16 cells were pre-inoculated intravenously into recipient mice. After 24 hours, mice in therapeutic group were treated peritoneally with 1 × 10^6^ DBA DC, while mice in control group were treated with PBS. The therapeutic DBA DC were injected every 7 days for three times. Survival rates of all mice were recorded and shown. (**c**) Photos of lung metastatic nodes of alloDC-treated and untreated mice pre-inoculated with 5 × 10^2^ B16 cells were taken. (**d**) Statistics of lung metastatic nodes of alloDC-treated and untreated mice pre-inoculated with 5 × 10^2^ B16 cells were shown. (**e**,**f**) Seven days after the 3^rd^ immunization, peripheral blood lymphocytes of treated and untreated mice were collected, whose proportions of KLRG1^+^CD8 T cells in CD8 T cells were compared. Each experiment represents 1 out of 3 experiments. n = 5. (**g**,**h**) 1 × 10^4^ B16 cells were injected intravenously into B6 mice. After 24 hours, mice in combination therapy group were firstly treated peritoneally with 1 × 10^6^ DBA DC and then with 200 µg anti-PD1 on day 7, day 14 and day 21 post tumor inoculation. Mice in only alloDC or anti-PD1 treatment group were treated with the single drug as mentioned above. B16 lung metastasis (**g**) and the survival rates (**h**) of all mice were recorded and shown. These experiments were repeated for 3 times. n = 5 for each repeat. P values indicated the statistical significance when comparing with each monotherapy group.

As alloDC vaccination was shown to be effective in antitumor response, we determined whether alloDC could be exploited as therapeutic vaccine in cancer therapy. As was shown in Fig. 6a, we pre-inoculated different doses of B16 cells intravenously into recipient mice to mimic different number of circulating tumor cells. After 24 hours, mice in therapeutic group were injected peritoneally with 1 × 10^6^ DBA DC every 7 days, whereas mice in control group were treated with PBS. After vaccination for the third time, all mice did not receive any therapeutic treatment until the survival rates of each group were evaluated. We found that when 5 × 10^2^ B16 cells were pre-injected, the survival time of treated mice was significantly longer than control mice (Fig. 6b). Lung metastatic melanoma nodes were shown (Fig. 6c) and the number of melanoma nodes was compared in the 5 × 10^2^ B16 cell injection group, demonstrating less metastatic nodes in alloDC treated mice (Fig. 6d). However, as the pre-inoculated tumor dose increased, the therapeutic effects of alloDC vaccination became less effective (Fig. 6b). It is well accepted that larger tumor burden induced accelerated deterioration of immune microenvironments^24,25^, which could not be easily reversed by alloDC-activation. We wondered if adequate activation of KLRG1^+^CD8 T cells in these mice was effectively triggered in mice with higher tumor burden. Further investigation showed that even in mice injected with 5 × 10^4^ melanoma cells, KLRG1^+^CD8 T cells could also expand in numbers as effectively as in mice with 5 × 10^2^ melanoma cells (Fig. 6e,f). As was shown in Fig. 3a, the number of KLRG1^+^CD8 T cells increased after alloDC activation and peaked at day 7~10. As was shown in Fig. 4, KLRG1^+^CD8 T cells expressed higher amounts of inhibitory molecules, such as Tim-3, Lag-3 and PD-1. We speculated that besides the relatively low E:T ratio in large tumor burdens, antitumor effects of KLRG1^+^CD8 T cells would also be repressed because of the interaction of their inhibitory molecules and the rapidly deteriorating tumor microenvironments^26^. To break down the inhibitory microenvironment, we tried to inject anti-PD1 in combination with alloDC in tumor treatment and the combination therapy displayed a more efficient antitumor capability than only anti-PD1 or alloDC injection (Fig. 6g,h). Therefore, we concluded that alloDC immunotherapy showed more effective antitumor effects in mice with fewer tumor cells, which also indicated the best timing for alloDC-elicited immunotherapy. Blockade of immune checkpoint molecules could facilitate alloDC-elicited tumor suppression, indicating a promising combination strategy for immunotherapy.

## Discussion

Similar with ipilimumab (anti-CTLA-4)^27,28^ and anti-PD-1^29^, which promotes antitumor immune responses extensively, allogeneic cells and allogeneic MHC molecules are detected to activate 1~10% T cells^17^ and thus also widely improve antitumor microenvironments. One point of view suggest that the reason why these non-specific activation methods could benefit antitumor effects is to elicit bystander tumor antigen specific CTL responses^8^. However, in our alloDC-vaccinated mice, tumor antigen specific CD8 T cells were not found to increase. Instead, a population of KLRG1 expressing CD8 T cells increased, whose potent cytotoxicity against multiple tumor cells suggested that this tumor killing was not TCR-dependent and antigen specific. Their expression of NK cell receptors (Fig. 4i) indicated NK-like recognition and cytotoxicity against tumor cells. Although previous studies on KLRG1^+^CD8 T cells showed that they were effector T cells which killed antigen-specific targets like CTLs with high granzyme expression^30,31^, the gene expression profile analysis of these cells showed that they were more like activated effector CD8 T cells with upregulated expression of some NK cell receptors, indicating that alloDC-vaccination activated KLRG1^+^CD8 T cells might utilize missing-self recognition to exert cytotoxicity against tumor cells. Combined with previous researches^30,31^, all the results suggested that KLRG1^+^CD8 T cells might exert cytotoxicity against target cells in both antigen-specific and non-antigen-specific manners, depending on the nature of stimulus.

The underlying mechanisms how allogeneic APCs could mediate alloreactivity of recipient T cells are still unclear. Immunologists have already proposed several possible allostimulation mechanisms: (1) allogeneic MHC molecules themselves could stimulate 1–10% of T cells^17^; (2) allogeneic DCs could firstly stimulate bystander host DCs, which then activate CD8 T cells^15,16^; (3) allogeneic DCs could help create a pro-inflammatory microenvironment with increased antitumor-related cytokines, which promote the non-specific activation of CD8 T cells^17^. Here we described the phenomenon that allogeneic dendritic cells could activate the increase of KLRG1 expression CD8 T cells, establishing the correlation between allostimulation and KLRG1^+^CD8 T cells. It may provide a new sight for a further investigation of the mechanisms by which allogenenic cells stimulate mixed lymphocyte reactions.

Although KLRG1 is considered as an immune checkpoint marker and anti-KLRG1 antibody has been shown to prevent tumor metastasis^32^, it is not contradictory with our findings that these KLRG1^+^CD8 T cells with exhaustion phenotypes could also exert antitumor effects. Theoretically, activated CD8 T cells can acquire immune checkpoint markers to suppress excessive stimulation of immune system so as to avoid autoimmune diseases. Studies on KLRG1 also demonstrated that KLRG1 expression might be a negative feedback to prevent autoreactivity^33^. Laboratory studies also showed that PD1^+^T cells had a higher frequency of tumor reactive T cells^34^. Our data (Fig. 4) presented that KLRG1^+^CD8 T cells also expressed a higher level of PD1. This indicated that these KLRG1^+^CD8 T cells might also be activated CD8 T cells with a higher frequency for tumor reactive T cells, which could inhibit tumor growth efficiently.

As to the KLRG1^+^CD8 T cells, previous researches mainly focus on their function as a subset of effector T cells. For example, KLRG1^+^CD8 T cells serve as short-lived effector CD8 T cells during viral infections^30^. Also, some researches show the KLRG1^+^CD8 T cells are senescent and exhausting effector cells^30,35^ while others suggest that presence of senescence markers does not mean loss of protective effector ability in these cells^36^. However, in our alloDC vaccination antitumor model, we found that these KLRG1^+^CD8 T cells could be activated by alloDC and survive for more than 28 days, indicating that these alloDC-induced KLRG1^+^CD8 T cells are not just senescent effector cells, but long lived effector cells. Since the marker KLRG1 is considered to be an inhibitory receptor which raise the activation threshold of KLRG1-expressing effector cells including T and NK cells and attenuate their effector responses^33^, we hypothesized that KLRG1^+^CD8 T cells could be recognized as an activated super killing population with more cytotoxic “weapons”^30,31^ and more inhibitory “supervision”^18,37,38^ to avoid overactivated cytotoxicity. Recently, immunologists have acquired great advances in harnessing immune checkpoint antibodies to block inhibitory receptors^39^, which suggested that KLRG1 might be the next target to prompt the cytotoxicity of KLRG1^+^CD8 T cells against tumor cells. However, considering the lack of study on KLRG1 signaling, how the KLRG1^+^CD8 T cells recognize the tumor cells and how the cytotoxicity of these cells is regulated, needs to be further studied.

The discovery of alloDC-induced KLRG1^+^CD8 T cells also indicated the presence of small subsets of cells which might play a key role in some immune responses. The immune system is composed of many types of immune cells, which make up the complicated but orderly defense network^40^. In individuals undergoing tumorigenesis, a large number of effector cells, including CTLs and NK cells^41^, are known to be recruited into tumor sites and impose restraints on tumor growth. However, the contributions of smaller subsets (e.g., KLRG1^+^CD8 T cells) are often overlooked because of the lack of practical research methods. Herein, we found that alloDC vaccination could lead to the increase of the small KLRG1^+^CD8 T cell subset, which was thoroughly ignored in allogeneic cells induced immune response previously. Our data also indicated that alloDC-vaccination could be used as therapeutic vaccines to eliminate residual circulating tumor cells so as to prevent tumor recurrence after conventional treatments for cancer patients. Although alloDCs showed a significant antitumor effects in different mouse models, we have to admit that from most of our experiments, alloDCs mainly exhibited a prophylactic effect rather than actual antitumor effect. Besides, the underlying mechanisms how allogeneic dendritic cells activated KLRG1-expressing CD8 T cells also remained to be further investigated, in order to get a deeper understanding of the interactions between allogeneic cells and effector CD8 T cell subsets.

## Methods

### Mice

B6 mice (H-2^b^) and β_2_m^−/−^ mice (B6.129P2-*B2m*^*tm1Unc*^/J) were obtained from Jackson Laboratory and were bred in specific pathogen-free conditions in Laboratory Animal Research Center, Tsinghua University (Beijing, China). DBA/2 mice (H-2^d^), Balb/c mice (H-2^d^), C3H mice (H-2^k^), FVB/NCrlVr mice (H-2^q^), and SJL/JOrlCrlVr mice (H-2^s^) were purchased from Vital River Laboratory Animal Technology Co. Ltd, Beijing, China. All mice were used at 6–8 weeks of age. The mice were treated in accordance with the National Institute of Health Guide for the Care and Use of Laboratory Animals with the approval of the Scientific Investigation Board of Tsinghua University (15-ZMH1), Beijing. B16-F10 and EL4 cell lines were purchased from ATCC. H22 and S180 cell lines were purchased from National Infrastructure of Cell Line Resource.

### Preparation of dendritic cells

Mature DCs from the different mouse strains were prepared from bone marrow according to the established method^42^. Unless indicated otherwise, DCs were intraperitoneally administered at 1 × 10^6^ cells per recipient mouse.

### *In vitro* cytotoxicity assay

EL4 thymoma cells were used as target cells, and were stained at 4 °C with CMFDA (Molecular Probes, Invitrogen) at a concentration of 1 μmol/10^6^ cells/mL. After 10-min incubation, cells were washed 3 times with PBS containing 10% FCS. Lymphocyte subsets were sorted by flow cytometry and co-cultured in 96-well plates with 1 × 10^4^ target cells in RMPI 1640 containing 10% FCS and 50 U/mL of recombinant IL-2 (R&D) at indicated E/T ratios. Cells were harvested every 12 h and were incubated with 7-AAD (Molecular Probes, Invitrogen) at room temperature for 10 min. The cells were then washed once with PBS and analyzed on a BD FACSAira II. The percentage of 7-AAD–positive cells indicated killing rates.

### Antibodies and flow cytometric analysis

To analyze lymphocyte subsets and assess expression of surface markers, lymphocytes were stained with monoclonal antibodies recognizing CD45.1, CD45.2, CD90.1, TCRβ, NK1.1, CD8, CD27, CD43, CD44, CD62L, CD127, ICAM-1, NKG2D (eBioscience) and NKG2A/C/E, CD16/CD32 (BD Pharmingen). Flow cytometry was performed using a FACSAria II (Becton Dickinson), and data were analyzed with FlowJo software.

### Sorting of different immunocytes

A total of 10^8^ splenocytes from DBA/2 mice or B6 mice were isolated, respectively and labeled with fluorescent antibodies to TCRβ, CD19, CD11c and Ia markers. Then 3 × 10^7^ T cells (TCRβ^+^CD19^−^), 5 × 10^7^ B cells (TCRβ^−^CD19^+^) as well as 1 × 10^6^ DCs (TCRβ^−^CD19^−^CD11c^+^Ia^+^) from both DBA/2 mice and B6 mice were sorted out by FACSAria II flow cytometry.

### CD8 T cell enrichment and adoptive transfer

B6 mice were intraperitoneally injected with PBS, 1 × 10^6^ B6 DCs or 1 × 10^6^ DBA DCs every two weeks. Three days after the second injection, recipient mice were sacrificed, and splenocytes were harvested. Splenic CD8 T cells were isolated by positive selection using magnetic beads conjugated to an antibody recognizing the CD8 molecule (Miltenyi Biotech) and injected intravenously into recipient mice.

### Characterization of tumor-infiltrated lymphocytes

Mice (n = 6/group) were immunized bi-weekly by intraperitoneal injection with 1 × 10^6^ B6 DC, 1 × 10^6^ DBA DC or PBS. Seven days after the second immunization, 5 × 10^5^ B16 cells were injected into the tail vein. On Day 14, mice were sacrificed and lungs were perfused using PBS. A piece of lung (9.8 ± 1.7 mg) was removed and then digested in 1 mg/mL collagenase IV (Sigma) at 37 °C for 1 h. Dissociated cells were collected through a 70-μm filter and stained with APC-Cy7 conjugated anti-CD45.2, FITC conjugated anti-TCRβ, PerCP conjugated anti-NK1.1, PE conjugated anti-CD8 and APC conjugated anti-KLRG1 antibodies (eBioscience). Flow cytometry (BD FASAria II) was used to collect data and the absolute number of KLRG1^+^CD8 T cells for each sample was calculated by adding counting beads (Spherotech) to each sample. CD45.2^+^ cells were gated and analyzed to characterize the tumor-infiltrated lymphocytes.

### RNA-seq analysis

1 × 10^6^ DBA DC or B6 DC were peritoneally injected into B6 mice for two times every 2 weeks. Three days after the second immunization, different populations of splenocytes were sorted by flow cytometry (BD FACSAria II). Each lymphocyte population has got the number of at least 5 × 10^6^ for RNA-seq. Then high throughput transcriptom sequencing was performed in Beijing Genomics Institute and differentially expressed genes (DEGs) were selected. Gene ontology enrichment, KEGG enrichment as well as protein-protein interaction network on DEGs was demonstrated using R ggplot2 package and Cytoscape software. Expression profiles of immunity-related genes, such as CD surface markers, interleukins, chemokine and chemokine receptors, and cytotoxicity-associated molecules, were also shown using R pheatmap package.

### Real-time PCR

Total RNA was extracted from indicated immunocytes using TRIzol (Invitrogen) and cDNA was further synthesized using Reverse Transcriptase Kit (Qiagen). Quantitative real-time PCR was performed using the Power Up SYBR Green Master Mix (ABI) to quantify the expression levels of genes (actin as the internal control). PCR with real-time fluorescence detection was performed on ABI StepOnePlus^TM^ (Applied Biosystems). Data analysis was performed using ABI Stepone software (Applied Biosystem). The specific primers were designed according to their corresponding CDS regions and listed in Supplementary Materials.

### Frozen section and immunohistochemistry

1 × 10^6^ B6 DC or 1 × 10^6^ DBA DC were intraperitoneally injected bi-weekly into recipient B6 mice. Seven days after the second vaccination, 5 × 10^5^ B16 cells were injected intravenously. On day 14, mice were sacrificed, and their spleens or B16-bearing lungs were harvested for frozen sections and immunohistochemistry analysis. The harvested lungs were embedded with SAKURA Tissue-Tek OCT compound and rapidly frozen to obtain serial 8-μm sections by Leica CM1900 cryostat. Sections were fixed in acetone and then incubated for 1 h at room temperature with PBS containing 1% rat serum. Afterwards, sections were stained for 15 min at 4 °C with PE-conjugated antibody to CD8, APC-conjugated antibody to KLRG1, and DAPI (Sigma). Sections were rinsed twice with PBS prior to placing the cover slip. Fluorescent antibody staining was evaluated by confocal microscopy using an Andor live cell spinning disk microscope and captured images were analyzed by the software Imaris, which could provide more intuitive images by transforming fluorescent dye-labeled cells into highlighted colored balls.

### *In vitro* matrigel invasion assay

1 × 10^5^ B16-GFP cells or EL4-GFP were cultured in the lower layer of the matrigel invasion chamber 24-well plate (Corning) for 24 hours. Then 2 × 10^5^ KLRG1^+^CD8 T cells or KLRG1^−^CD8 T cells sorted from splenocytes of DBA DC-vaccinated mice were added into the upper layer of the chambers. After 12 hours, GFP negative cells in the lower layer were collected, whose absolute number was calculated using counting beads (Spherotech) by flow cytometry.

### Live cell imaging

KLRG1^+^CD8 T cells and KLRG1^−^CD8 T cells were sorted from DBA DC vaccinated mice. Then they were co-cultured with B16-GFP cells (green) at the E:T ratio of 3:1 or 5:1. A dynamic display of KLRG1^+^CD8 T cell-mediated killing process was captured using PE spinning disk live cell confocal microscopy with a 60 × oil immersion lens.

### Statistics

A two-tailed Student’s t-test was used to compare 2 groups of normally distributed samples, and a Mann-Whitney U test was used when 2 groups of abnormally distributed samples were compared. The cox regression model was used to evaluate survival times between 2 groups. Error bars show standard errors. Different between groups could be judged as statistically significant when P < 0.05 or less. ^***^Means P < 0.001, ^**^means P < 0.01, ^*^means P < 0.05 and “ns” means not significant.

## Supplementary information

Supplementary Figures

Supplementary Video 1

Supplementary Video 2
