# Supplementary material for: Allogeneic dendritic cells induce potent antitumor immunity by activating KLRG1+CD8 T cells
Source: Sci Rep. 2019 Oct 29;9:15527. doi: 10.1038/s41598-019-52151-3 (PMC6820535; doi:10.1038/s41598-019-52151-3)
Supplement: Supplementary file 1 — Supplementary Figures [file 41598_2019_52151_MOESM1_ESM.pdf]

**Supplementary Materials:**

# Allogeneic dendritic cells induce potent antitumor immunity by activating KLRG1$^+$CD8 T cells

Chao Wang[1,4,6], Zhengyuan Li[1,6], Zhongli Zhu[2], Yijie Chai[1], Yiqing Wu[1], Zhenglong Yuan[1], Zhijie Chang[3,7], Zhao Wang[4,7], Minghui Zhang[1,5,7]

[1]School of Medicine, Tsinghua University, Beijing 100084, China

[2]Clinical Laboratory, Affiliated Hospital of Taishan Medical University, Taian, Shandong 271000, China

[3]State Key Laboratory of Biomembrane and Membrane Biotechnology, School of Medicine, School of Life Sciences, Tsinghua University, Beijing 100084, China

[4]MOE Key Laboratory of Protein Sciences, School of Medicine, Tsinghua University, Beijing 100084, China

[5]The Central Laboratory, The First Hospital of Tsinghua University, Beijing 100084, China

[6]These authors contributed equally to this work.

[7]Correspondence should be addressed to Zhijie Chang, Zhao Wang & Minghui Zhang:

Zhijie Chang: School of Medicine, School of Life Sciences, Tsinghua University, Beijing 100084, China. Email: zhijiec@tsinghua.edu.cn; Tel: 86-10-62799520

Zhao Wang: Tsinghua University, Beijing 100084, China. Email: zwang@tsinghua.edu.cn; Tel: 86-10-62799520

Minghui Zhang: School of Medicine, Tsinghua University, Beijing 100084, China. Email: mh-zhang@mail.tsinghua.edu.cn; Tel: 86-10-62799520

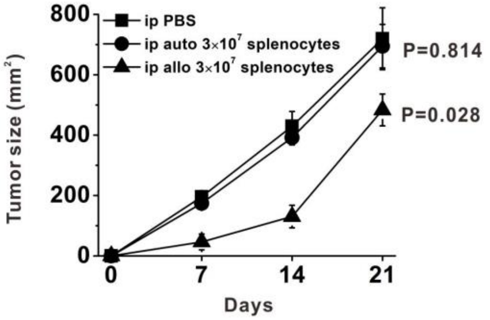


**Supplementary Fig 1.**

Vaccination with $3\times10^7$ allogeneic splenocytes could prevent EL4 tumor growth. B6 mice were pre-immunized with $3\times10^7$ splenocytes from DBA/2 mice (▲) or B6 mice (●), respectively. After immunization for two times, they were inoculated subcutaneously with $2\times10^6$ EL4 cells. EL4 tumor sizes were detected at indicated time points. P values indicated the statistical significance when comparing with blank control group (ip PBS group).

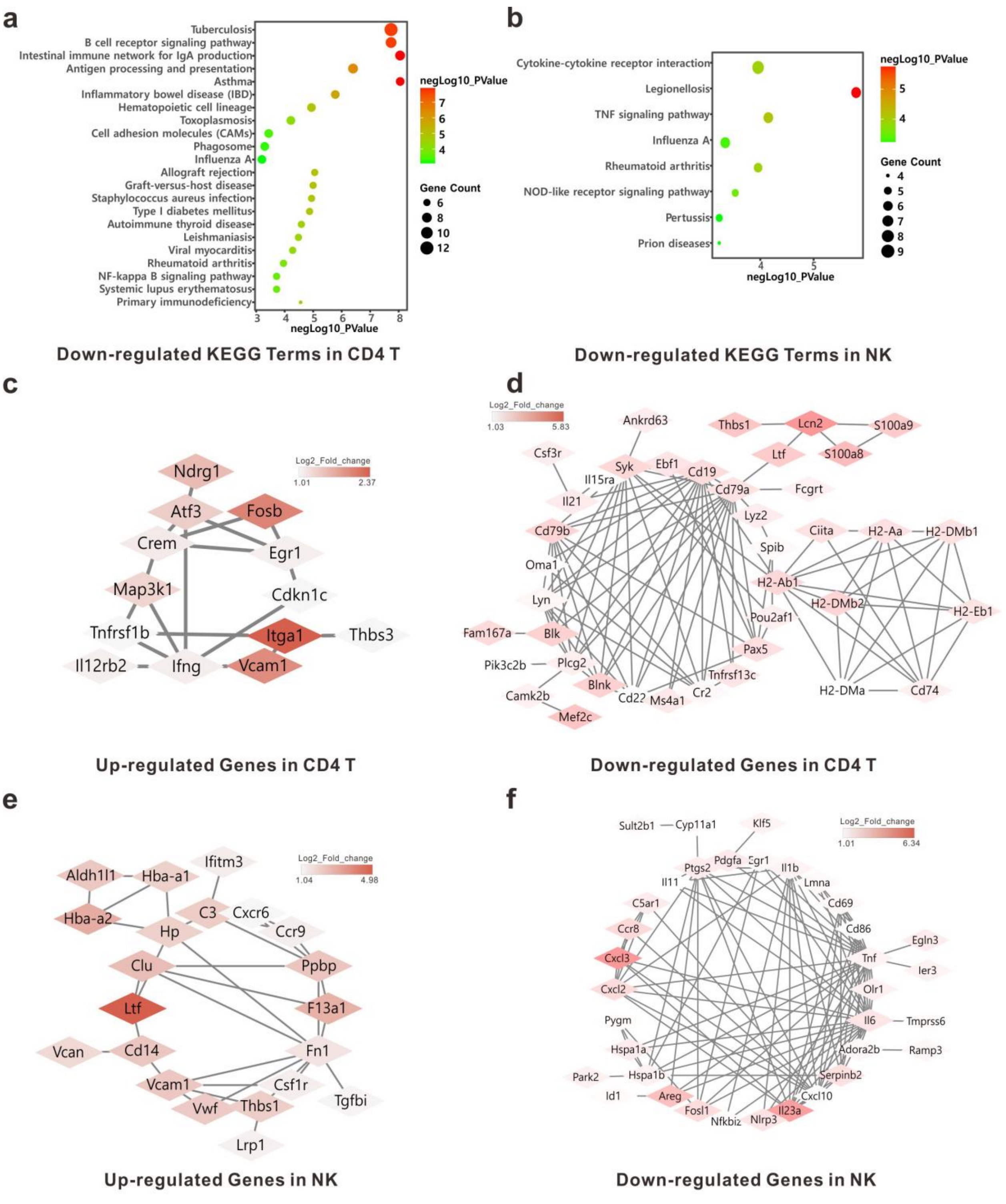


**Supplementary Figure 2.**

Transcriptomic features of CD4 T cells and NK cells in alloDC-vaccinated mice. RNAseq on CD4 T cells or NK cells from autoDC- and alloDC-vaccinated mice was performed and their respective DEGs were analyzed by KEGG pathway enrichment (a,b) and protein-protein interaction network analysis (c-f).
